# First Evidence of Kv3.1b Potassium Channel Subtype Expression during Neuronal Serotonergic 1C11 Cell Line Development

**DOI:** 10.3390/ijms21197175

**Published:** 2020-09-29

**Authors:** Hager Tabka, Amani Cheikh, Sonia Maatoug, Mohamed El Ayeb, Saïd Bendahhou, Rym Benkhalifa

**Affiliations:** 1 Laboratoire Venins et Molécules Thérapeutiques, Institut Pasteur de Tunis, Tunis El Manar University, 13 Place Pasteur BP74, 1002 Tunis, Tunisia; cheikhamani@gmail.com (A.C.); soniamaatoug@hotmail.fr (S.M.); mohamedelayeb21@gmail.com (M.E.A.); 2 Faculty of Sciences of Bizerte, Carthage University, 7021 Bizerte, Tunisia; 3 Faculté de Médecine, Université Côte d’Azur, UMR7370 CNRS, LP2M, Labex ICST, Nice, France; said.bendahhou@univ-cotedazur.fr

**Keywords:** Kv3.1 channel, 1C11 cell line, fluoxetine, 5HT receptors, serotonin dosage

## Abstract

Kv3.1 channel is abundantly expressed in neurons and its dysfunction causes sleep loss, neurodegenerative diseases and depression. Fluoxetine, a serotonin selective reuptake inhibitor commonly used to treat depression, acts also on Kv3.1. To define the relationship between Kv3.1 and serotonin receptors (SR) pharmacological modulation, we showed that 1C11, a serotonergic cell line, expresses different voltage gated potassium (VGK) channels subtypes in the presence (differentiated cells (1C11D)) or absence (not differentiated cells (1C11ND)) of induction. Only Kv1.2 and Kv3.1 transcripts increase even if the level of Kv3.1b transcripts is highest in 1C11D and, after fluoxetine, in 1C11ND but decreases in 1C11D. The Kv3.1 channel protein is expressed in 1C11ND and 1C11D but is enhanced by fluoxetine only in 1C11D. Whole cell measurements confirm that 1C11 cells express (VGK) currents, increasing sequentially as a function of cell development. Moreover, SR 5HT1b is highly expressed in 1C11D but fluoxetine increases the level of transcript in 1C11ND and significantly decreases it in 1C11D. Serotonin dosage shows that fluoxetine at 10 nM blocks serotonin reuptake in 1C11ND but slows down its release when cells are differentiated through a decrease of 5HT1b receptors density. We provide the first experimental evidence that 1C11 expresses Kv3.1b, which confirms its major role during differentiation. Cells respond to the fluoxetine effect by upregulating Kv3.1b expression. On the other hand, the possible relationship between the fluoxetine effect on the kinetics of 5HT1b differentiation and Kv3.1bexpression, would suggest the Kv3.1b channel as a target of an antidepressant drug as well as it was suggested for 5HT1b.

## 1. Introduction

In the brain, the alternating activation of different ionic currents establishes the resting membrane potential, generates the action potential and regulates the neuronal firing frequency and neurotransmitter release, thus providing neuronal cells with specific electrical identity [1,2]. The voltage-dependent Kv3 family generates such high frequency activity because they are important in neuronal excitability and plasticity [3].

The Kv3 channels family presents four subtypes—Kv3.1, Kv3.2, Kv3.3, and Kv3.4—that are distinguished from the other mammalian Kv families by their typical opening at positive potentials and by their rapid activation and deactivation kinetics [4,5,6,7]. Otherwise, the Kv3.1 channel subtype is highly sensitive to 4-aminopyridine (4-AP) and to tetraethylammonium TEA [6,8] and is inhibited by fluoxetine, an antidepressant drug known as Prozac [9].

In the brain, Kv3.1 is abundantly expressed in neurons that are able to fire at high frequencies, like cerebellar granule cells [10,11] and some inhibitory interneurons [12]. Previous studies done in mouse hemibrains at different stages, revealed that all Kv3 transcripts were significantly expressed in the embryonic age and this expression increases progressively [13]. There are two subtypes of Kv3.1 -a and –b, which differ in expression during development. Kv3.1a is expressed in the neurons of the adult brain whereas Kv3.1b is expressed in embryonic and perinatal neurons [14]. Numerous studies highlighted the growing number of hereditary or acquired diseases with which this channel is associated. Kv3.1 is involved in neurologic epilepsy [15] and neurodegenerative diseases such as multiple sclerosis [16] and Alzheimer’s [13], in cancer tumor hypoxia [17] and in behavior disorders such as bipolar disorder [18] and schizophrenia [19]. Kv3.1 dysfunction was also reported in cases of circadian cycle disturbance, sleep loss [20,21] and depression [22].

Most neuropsychiatric diseases, including schizophrenia and depression, are currently treated with medications that have a high affinity for serotonin receptors 5HT [23]. In fact, during depression, selective serotonin reuptake inhibitors like fluoxetine (SSRIs) are the most commonly prescribed drugs [24].

Fluoxetine interacts with the channel open state [9] and blocks, at micromolar concentrations, several potassium channel subtypes, as Kv1.1, Kv1.3, Kv1.4, Kv1.5, Kv3.1, Kv4.3, hERG and TREK-1 [25,26,27,28].

We have previously demonstrated the presence of a peptidic inhibitor in *Androctonus australis hector* scorpion venom [29] active on the Kv3.1b channel and running data conduct the biochemical and pharmacological characterization of this bioactive component (data not shown). Moreover, a recent study reports that changes in neuronal cells activity during acute and/or chronic SSRI treatment correlates with the changes in the function of the Kv3.1 channel. In neuronal circuits, Kv3.1 is differentially regulated: antipsychotic treatment elevates the Kv3.1 level in the cortex but, in the hippocampus, chronic antidepressant drug use resulted in reduced activity of this channel [30].

For these reasons, we propose in this study to define the relationship between the expression of the Kv3.1b and the serotonergic activity of the 1C11 cell line, using fluoxetine, their common modulator. 1C11 is a murine serotonergic cell line from neuronal stem cells and may undergo either serotoninergic or noradrenergic differentiation upon induction [31].

We suggest also to determine whether and how the cell line 1C11 expresses the Kv3.1 channel during cell proliferation and differentiation. We therefore compared the fluoxetine impact on 5HT1b expression versus Kv3.1 by RNA quantification and the rate of protein expression.

We further demonstrated, in vitro on the neuronal serotonergic cells line 1C11, that (1) the Kv3.1b channel is significantly expressed, (2) fluoxetine affects Kv3.1b expression but increases cell proliferation and enhances the expression of 5HT1b even in the absence of precursors and (3) Kv3.1b expression depends on the cell differentiation stage.

## 2. Results

### 2.1. Evaluation of Kv3.1b Gene Expression in a 1C11 Cell Line

#### 2.1.1. Kv3.1b Gene Expression in 1C11

1C11 cells have the ability to secrete serotonin after differentiation thanks to 5HT receptors. This study was designed to determine whether Kv3.1b channel activity is related to the 1C11 serotonergic activity. In vitro, 1C11 cells proliferate in two steps: (i) they divide until confluency and (ii) under the precursor’s application, they differentiate by expressing 5HT receptors; in addition, cells can self-differentiate.

We first verified the expression of the Kv3.1.b channel gene in 1C11 cells by RT-PCR analysis.

**Figure 1:**
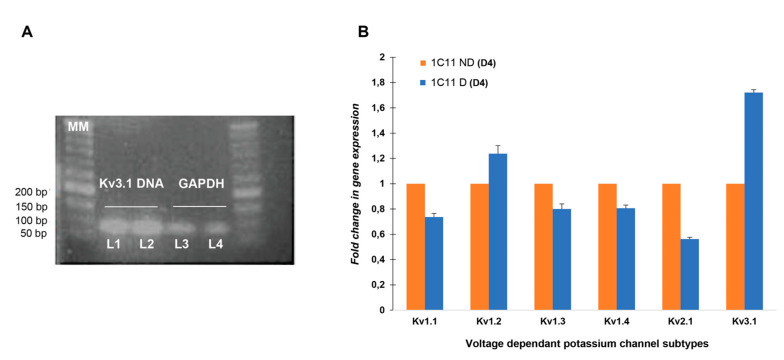
(**A**). The gel electrophoresis of Kv3.1b using Kv3.1 and 2 primers for the characterization of the expression of kv3.1b, isolated from 1C11 serotonergic neuronal stem cells. (MM) Molecular weight marker. Lane 1:Kv3.1b in 1C11ND(D4) cells; Lane 2: Kv3.1b in 1C11D(D4) cells; Lane 3 and 4: GAPDH (Positive control). (**B**). Kv subtypes mRNA quantification in 1C11 measured with qRT-PCR. 1C11ND(D4), not differentiated cells; 1C11 D(D4), differentiated cells (*n* = 3). Fold change in gene expression is calculated through the 2 ΔΔCT method [32]. Data from 3 different independent cultured 1C11 cell line, with 3 replicates for each condition (1C11ND and 1C11D), Analysis by a Student’s *t*-test for unpaired data was used to compare the means between two populations of Kv1.1, Kv1.2, Kv1.3, Kv1.4, Kv2.1 and Kv3.1 channels, in the presence or absence of induction. *p* < 0.05.

The gel in Figure 1A shows that PCR products were displayed at 100 bp size, as expected, which suggests that the neuronal stem cell clones of 1C11 expressed the Kv3.1.b channel mRNA in cells in the presence or absence of induction. Since cell excitability is dependent on different kinds of potassium channel activity, we attempted to identify, under the same experimental conditions, the expression level of those known to be present in neurosecretory cells, such as Kv1.1, Kv1.2, Kv1.3, Kv1.4 and Kv2.1 besides Kv3.1 mRNA.

#### 2.1.2. Quantification of Kv3.1 Besides Kv1.1, Kv1.2, Kv1.3, Kv1.4 and Kv2.1 mRNA Expression in 1C11

We used real-time quantitative PCR (qPCR) in pools of 1C11 cell lines for a more quantitative analysis of mRNA expression. The relative quantification of Kv3.1 RNA is normalized to the GAPDH gene using the 2^−ΔΔCT^ method [33].

Figure 1B histograms show the real-time PCR analysis of several Kv channel transcripts expression: Kv1.1, Kv1.2, Kv1.3, Kv1.4, Kv2.1 and Kv3.1, in 1C11ND(D4) as well as in differentiated cells 1C11D(D4) (Figure 1B).

In 1C11ND(D4) cells, the different Kv channels, either delayed rectifier or Shaw transcript subtypes, show the same level of expression (1 ± 0). Only during differentiation did the expression of the Kv1.2 (1.237 ± 0.063) and Kv3.1.b (1.720 ± 0.022) transcript increase considerably with the highest transcripts level in the Kv3.1.b subtype. It is also noteworthy that the expression of Kv1.1 (0.737 ± 0.028), Kv1.3 (0.80 ± 0.039), Kv1.4 (0.806 ± 0.025) and Kv2.1 (0.562 ± 0.013) is significantly downregulated (*p* < 0.05).

It thus appears that the different channel subtypes contribute in the same way to generating Kv currents, but a remarkable presence of Kv3.1.b in differentiated cells suggests that it is very involved in the cell’s differentiation mechanism in mature cells.

### 2.2. Potassium Channel Expression During 1C11 Development in the Culture

In vitro, seeded cells adhere to the plate, and proliferate by dividing and after differentiation. During proliferation, cells develop ramifications while dividing, which makes the induction of spontaneous differentiation. In experimental conditions, when cells are at 80% confluence, adding precursors can enhance the differentiation phase.

In our experimental conditions, global potassium currents were recorded in 1C11 cells during proliferation and after induction.

**Figure 2:**
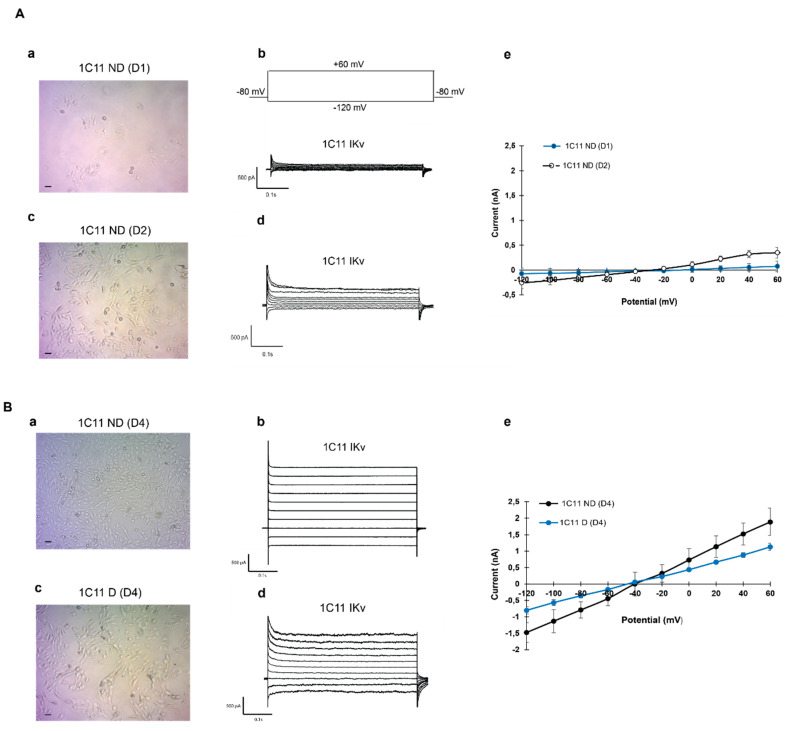
Whole cell potassium channels current recordings in 1C11 cell lines (**A**) 1C11ND(D1) are round (**a**) on day 1 and present a minority potassium current: IKv (**b**). 1C11ND(D2) developing little ramification in day 2 (**c**) shows a higher current amplitude under the same conditions (**d**). Current–potential relationships in 1C11ND(D2) cells are illustrated in (**e**). (**B**) 1C11ND(D4) cells on day 4 (**a**) show the highest potassium current amplitude (**b**). The micrography (**c**) presents differentiated cells1C11D(D4) in the presence of precursors cultured in the same conditions and with potassium current amplitude two times less important (**d**). I–V relationships are illustrated in (**e**). Data from (3–6) different independent cultured 1C11 cell line (1C11ND D1, 1C11D D2, 1C11ND D4 and 1C11D D4). Analysis by a Student’s *t*-test for unpaired data is used to compare the means between two populations of the potassium current amplitude in the presence or absence of induction during culturing. *p* < 0.05.

During proliferation, 1 day after cells seed, round cells show small potassium current amplitudes of about 0.073 ± 0.007 Na, whereas cells with short ramifications had a higher current amplitude (0.347 ± 0.045 nA) as shown in Figure 2A. In mature cells, even if the differentiation was spontaneous or enhanced with precursors, the amplitude of the potassium current increased significantly. In mature cells, IKv was 1.05 ± 0.10 nA in the presence of precursors and reached 1.77 ± 0.10 nA when cells were confluent, see Figure 2B (*n* = 7–11).

### 2.3. Variation of Kv3.1b and 5HT1b mRNA Expression in the 1C11 Cell Culture In Vitro And As A Function of Fluoxetine

As previously mentioned, the serotonergic 1C11 cell line did not express 5HT receptors before the induction contrary to differentiated cells. The antidepressant molecule, fluoxetine, inhibited both the serotonin reuptake capture and the Kv3.1.b channel current in the 1C11 cell line. These experiments concerned the study of the effect of fluoxetine on 5HT receptors and Kv3.1 voltage gated channel transcript expression in 1C11 cells before and after differentiation.

#### 2.3.1. Analysis of the Fluoxetine Effect on 5HT1b Expression In the 1C11 Cell Line

**Figure 3:**
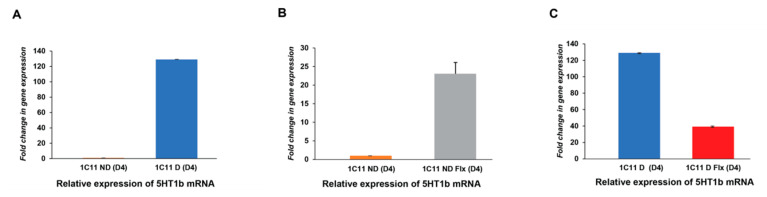
Fluoxetine effect on 5HT1b mRNA levels in the 1C11 cell line during culture. The 5HT1b mRNA level is determined by qRT-PCR. (**A**) Real time PCR analysis confirms the high expression of 5HT1b receptors in 1C11 differentiated cells, contrary to proliferating cells. 1C11 ND (cells in proliferation) and 1C11 D (differentiated cells 1C11-5HT). (**B**). Fluoxetine improves the mRNA expression level of 5HT1b in ND1C11 but drastically decreases it in differentiated cells. (**C**) 1C11D (Flx; 1C11-5HT + FLX 10 nM) and 1C11 ND (Flx; 1C11 + FLX 10 nM; *n* = 3). Fold change in gene expression is calculated using the 2^ΔΔCT^ method. Data from 3 different independent cultured 1C11 cell line, with 3 replicates for each condition (1C11ND, 1C11D, 1C11NDFlx and 1C11DFlx). An analysis by an unpaired Student’s *t*-test is used to compare the means between two populations of 5HT1b receptors in the control condition or in the presence of fluoxetine. *p* < 0.05.

As expected, the quantitative analysis of the serotonin receptor 5HT1b shows a very weak mRNA expression in not differentiated cells (1C11ND (D4)) with N1_ND_ = 1 ± 0 and of 129.063 ± 0.133 in differentiated cells (1C11D D4; Figure 3A). Fluoxetine 10 nM application on 1C11 cells during proliferation increased the level of 5HT1b transcripts significantly to a value equal to N2_NDFlx_ = 23.062 ± 3.040 1C11 but decreased mRNA level to 39.336 ± 0.701 in differentiated cells (Figure 3B).

#### 2.3.2. Analysis of the Fluoxetine Effect on Kv3.1b Expression in the 1C11 Cell Line

**Figure 4:**
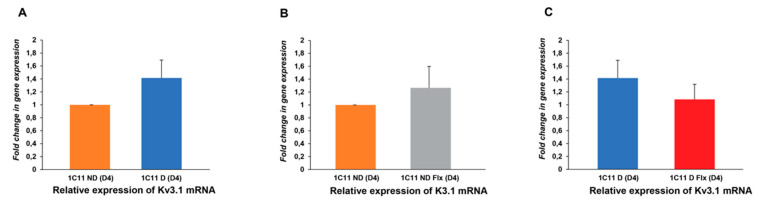
Fluoxetine effect on the Kv3.1b mRNA level in the 1C11 cell line in the culture. The Kv3.1b mRNA level is measured with qRT-PCR. (**A**) The histograms show a significant increase of the Kv3.1b mRNA level in differentiated cells compared to not-differentiated cells. Fluoxetine increases mRNA transcripts in culturing cells 1C11ND(D4) (**B**) but significantly decreases the mRNA level in differentiated cells (**C**). 1C11ND(D4) (cells in culture) and 1C11 D (differentiated cells 1C115-HT(D4))-. 1C11DFl × (1C11-5HT + FLX 10 nM(D4) and 1C11 NDFlx (1C11ND + FLX10 nM(D4); *n* = 3). Fold change in gene expression is calculated using the 2^ΔΔCT^ method [34]. Data from 3 different independent cultured 1C11 cell lines, with 3 replicates for each condition (1C11ND, 1C11D, 1C11NDFlx and 1C11DFlx). An analysis by unpaired Student’s t-test is used to compare the means between two populations of Kv3.1b channels in the control condition or in the presence of fluoxetine. *p* < 0.05.

The Figure 4A histograms show that the level of Kv3.1b expression in differentiated cells N1_D_ = 1.41 ± 0.27 was significantly higher than in not differentiated cells N1_ND_ = 1 ± 0 (Figure 4A). In the presence of fluoxetine, the expression level was affected in both cases. The level of Kv3.1b transcripts increased during culture cells N2_ND_ = 1.41 ± 0.27 and decreased in 5HT1b-cells N2_D_= 1.02 ± 0.23 (Figure 4B).

### 2.4. Evaluation of Kv3.1b Channel Protein Expression in Cultured 1C11

Both 5HT receptors and Kv3.1 channels were modulated with fluoxetine, which inhibited IKv3.1 by pore obstruction at a micromolar concentration and also inhibit serotonin reuptake and saturates 5HT subsites at a nanomolar concentration. We are seeking to highlight the Kv3.1b protein expression in 1C11 cell lines and to ascertain whether its expression is affected during fluoxetine application in differentiated versus not differentiated cells.

**Figure 5:**
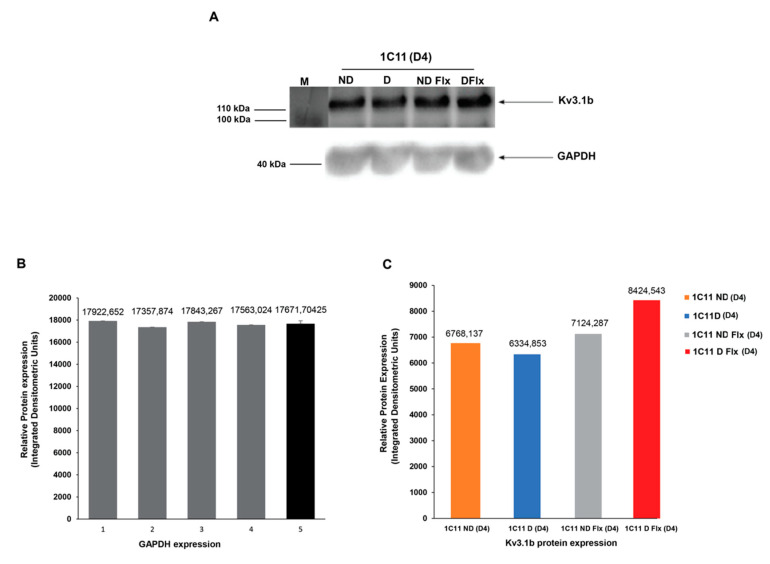
Analysis of Kv3.1b proteins expression in the 1C11 cell line in vitro. (**A**). Representative Western blots of Kv3.1b in not-differentiated (1C11ND(D4)) and differentiated (1C11D(D4)) cells in standard conditions and in the presence of fluoxetine (NDFlx 10 nM(D4) and DFlx 10 nM(D4)). (**B**). Densitometric quantification of Kv3.1b of the WB obtained bands in the same conditions by using GAPDH as a control. GADPH expression varies from 17357.65 to 17922.65 with an average of 17,671.70 ± 259.94 (*n* = 4). (**C**). Kv3.1b protein expression is the same in not-differentiated and differentiated 1C11 cells but fluoxetine increases expression in differentiated cells only.

The Western blot test shows the Kv3.1b expression in differentiated and not-differentiated cells and in the presence or not of fluoxetine. It confirms the presence of a specific Kv3.1b band at about 110 kDa in the cell lysates in all conditions (Figure 5A). In fact, the level of Kv3.1b protein expression was the same whether or not cells were differentiated.

### 2.5. Evaluation of the Fluoxetine Effect on the Serotoninergic Activity of 1C11 Cells

However, in the presence of fluoxetine, the expression level shows a significant increase in differentiated cells compared to not-differentiated ones.

These results indicate that fluoxetine seems to affect the expression level of the Kv3.1b channel primarily in differentiated 1C11 cells by enhancing membrane channel expression.

1C11 cells convert within 4 days to serotonergic cells that can metabolize, store and take up serotonin (5-HT) and express 5-HT1B/D, 5-HT2A and 5-HT2B receptors [35].

**Figure 6:**
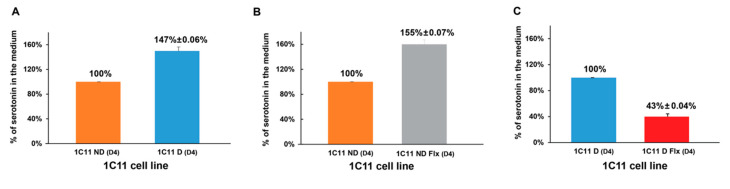
Dosage of serotonin in 1C11 cells in vitro. (**A**): Serotonin is higher when 1C11 cells are differentiated (D4; 147% ± 0.06%) contrary to not differentiated cells (D4; 100%). (**B**): Fluoxetine increased serotonin secretion of about 155% ± 0.07% in not-differentiated cells and decreased it by about 43% ± 0.04% (**C**). Data from 3 different independent cultured 1C11 cell lines for each condition (1C11ND, 1C11D, 1C11NDFlx and 1C11DFlx). An analysis by an unpaired Student’s *t*-test is used to compare the means between two populations’ 5HT1b receptors in the control condition or in the presence of fluoxetine. *p* < 0.05.

Figure 6 histograms show that, differentiated cells 1C11D(D4) released higher amounts of serotonin in the medium (5.8 ng/mL ± 0.36) than not-differentiated cells 1C11ND(D4). (3 ng/mL ± 0,4). When fluoxetine was added to the culture medium, the not-differentiated cells 1C11NDFlx(D4) released more serotonin (4.63 ng/mL ± 0.25) while differentiated cells 1C11DFlx(D4) show less serotonin being released (3.25 ng/mL ± 0.21).

## 3. Materials and Methods

### 3.1. Cell Culture and Transfection

A serotonergic cell line, 1C11, was used to study the effect of fluoxetine on Kv3.1b expression under different conditions. In addition to 1C11 the CHO cell line heterologously expressing the same potassium channel subtype was used as a positive control.

#### 3.1.1. C11 Cell Line

The neuronal stem cell clones 1C11 were provided by the Faculty of Medicine Paris Descartes Laboratory of Pharmacology and Cell Signaling—UMR-S 1124, team Toxicology, after MTA improvement.

The 1C11 cell line originates from immortalized precursor cells obtained from a multipotential embryonal carcinoma (EC) cell line transfected by a recombinant plasmid PK4 containing the early genes of SV40 under the control of the adenovirus ElA promoter [36].

The resulting immortalized cell lines 1C11 have the properties of committed stem cells that are able to differentiate further along a restricted lineage [37,38].

During the kinetics of the 5-hydroxytryptaminergic differentiation of the inducible 1C11 cell line, three different 5-HT receptors become detectable. The 5-HT1b receptor [32] expresses 2 days after the addition of db cyclic AMP and cyclohexane carboxylic acid (CCA1C11 cells were cultured in DMEM (Dulbecco’s modified Eagle’s medium; Gibco™, Sigma, Lyon, France) supplemented with 10% fetal bovine serum (Biochrom ref n° S011, Berlin, Germany). Cells were maintained at 37 °C in a humid atmosphere of 5% CO_2_. 1C11 cells were grown and induced to differentiate in the presence of 1 mM db cyclic AMP and 0.05% CCA [35]. Dibutyryl cyclic AMP (db cyclic AMP) and CCA were from Sigma-Aldrich (Lyon, France).

#### 3.1.2. Cell Treatment

1C11 cells were cultured in different conditions of differentiation (see Table 1).

#### 3.1.3. CHO Cell Culture and Transfection

The CHO, a cell line derived from Chinese hamster ovaries, was used as a positive control. CHO cells were routinely cultured in DMEM/F-12 (Lonza Bioscience, Portsmouth, NH, USA) supplemented with 10% FBS (Gemini Bio-products, West Sacramento, CA, USA) and 2 mM L-glutamine (Invitrogen, Carlsbad, CA, USA). Cells were maintained and passaged when they reached 80% confluence. Cells were transfected with the Kv3.1 DNAc plasmid with MaxCyte STX^®^ Scalable Transfection System (MaxCyte, Gaithersburg, MD, USA) to achieve a consistent level of expression. Plasmid containing EGFP was cotransfected to identify transfection-positive cells as a marker. Transfected cells were incubated for 24 h before being used in experiments.

### 3.2. Molecular Biology

#### 3.2.1. RNA Extraction, RT-PCR and Real-Time PCR

Total RNA was obtained from cultured cells. After RNA reverse transcription, real-time PCR was performed. Samples were normalized to the expression level of Kv3.1b or 5HT1b in 1C11 ND(D4), 1C11D(D4) and to the housekeeping gene *GAPDH*.

##### RNAs Extraction

The 1C11 cell lines were cultured for 4 days and all RNA was extracted using the RNeasy Mini Kit (Qiagen, Hilden, UK).

At 5 × 10^6^ densities, the cells were collected and treated with 1 volume of acetone-ethanol (1:1). The cells were left for 20 min in an ice bath or stored at −80 °C. On the day of the experiment, the cell suspension in acetone-ethanol was washed once in TSE and then resuspended in the same buffer. After enzymatic or mechanical lyses, as described above, 350 mL of RLP buffer (Qiagen, Hilden, UK) containing 14.4 M 2-mercaptoethanol (Amershan Biosciences, Munich, Germany) was added to each 200-mL cell lysate, which. was then placed in a microtube mixer for 30 min at 4000 rpm (Marconi, Brazil) at room temperature The material was then quickly frozen (ethanol-dry ice) and thawed (water bath at 60 °C) three times. Lastly, the RNA was purified using the RNeasy Mini Kit as per the manufacturer’s specifications for cells.

To ensure that the total RNA from the 1C11cells was free of DNA, the first treatment with DNase I was performed during the RNA clean up with the RNeasy Mini Kit following the manufacturer’s instructions (Qiagen, Hilden, UK). A second treatment with DNase I was performed after eluting the purified RNA from the column, as recommended by the manufacturer (Invitrogen, Carlsbad, CA, USA). The RNA preparation was stored at −80 °C.

Total RNA was quantified using the Nanodrop 1000 TM (Thermo Scientific, Waltham, MA, USA).

The quality of the RNA extracts was analyzed by electrophoresis on agarose 1.2% in 1 TAE (20 mM Tris acetate, 0.5 mM EDTA, pH 8.0) run at 110 V for 50 min. The gel was treated with ethidium bromide and visualized in a gel capture system (DNR Bio-Imaging System, Neve Yamin, Israel).

##### Reverse Transcription and PCR Amplification

Total RNA (1 μg) from each cell line was reverse-transcribed to cDNA using MMLV (Moloney Murine Leukemia Virus) reverse transcriptase (GeneOn, Ludwigshafen, Germany) as per the manufacturer’s instructions. The reverse transcription (RT) step involved incubation for 15 min at 42 °C and inactivation of the enzyme carried out for 10 min at 70 °C. The cDNA was stored at −20 °C. The polymerase chain reaction (PCR) was performed in a final volume of 50 μL of the reaction mixture containing 0.5 µg of cDNA; 20 picomoles of each primer corresponding to both ends of the target DNA (Table 1); 10 mM dCTP; 10 mM dATP; 10 mM dTTP 10 mM dGTP; 1× Taq polymerase buffer (50 mM KCl, 10 mM pH 8.3 and 1.5 mM MgCl_2_) and 1 unit of Taq polymerase (Amersham-Pharmacia, Munich, Germany). The thermal cycling conditions included an initial denaturation step at 95 °C for 5 min, followed by 35 cycles at 95 °C for 30 s, 58 °C for 30 s and 72 °C for 30 s. The glyceraldehyde 3–phosphate dehydrogenase (GAPDH) was used as a positive control. The primer sequences used for PCR and qPCR reactions are described in Table 2.

##### Quantitative-PCR

QPCR assays were performed on Roche’s the LightCycler^®^480 II System using the LightCycler^®^480 SW 1.5 Software. Brilliant II SYBR_Green QPCR Master Mix (Agilent, Santa Clara, CA, USA) was used for the qPCR reaction in a final volume of 25 μL.

The experimental reaction was prepared as recommended by the manufacturer. The primer sequences used are presented in Table 2. The endogen control GAPDH was prepared under the same conditions. The alternative protocol with three-step cycling cited in the manufacturer instructions was used with an initial denaturation step of 10 min at 95 °C followed by 40 cycles. Each cycle comprised of 3 steps: the DNA denaturation step at 95 °C for 30 s, a specific hybridization of the primers for 1 min at 58 °C for Kv3.1b primer and a polymerization at 72 °C for 30 s. Primer pair specificity was tested by amplification of the target using 10 ng of DNA.

A sample is considered positive if a specific signal is generated with a SYBR_Green qPCR method. Each assay was performed 3 times (*n* = 3).

We used the Kv3.1b gene in 1C11 ND (Kv3.1 ND) each time. The difference of the Ct is therefore equal to: ΔCt (sample) = Ct (gene of interest) − Ct (Kv3.1 ND). The result was then reported to a calibrator: GADPH (considered to be normal expression of the gene, for example in the case of a control which gives: ΔΔCt = ΔCt (sample) − ΔCt (calibrator). The amount of amplified template doubling at each PCR cycle, the relative expression of the gene ultimately calculated by the formula: 2^−ΔΔCt^.

#### 3.2.2. Protein Extraction and Western Blotting

Western blotting analyses were performed with the total protein obtained from the cultured cells, either 1C11 or CHO—transfected or not—cell lines. Protein extracts were collected by centrifugation at 11,000*g*. Cells collected after centrifugation were mixed with 5 mL of a 100 mM Tris–HCl buffer (pH 7.5) containing 0.5% (*w/v*) SDS and 0.25% (*w/v*) DTT. To accelerate the extraction, a high intensity focused ultrasounds (HIFU) probe (model VCX130, Sonics Vibra-Cell, Hartford, CT, USA) was used for 1 min at 30% of amplitude (corresponding to 69 V) followed by centrifugation (10 min, 4000× *g*). Proteins in the supernatant were precipitated with cold acetone (15 mL, 4 °C, 1 h) and centrifuged (10 min, 4000× *g*). The resulting pellet was dried at room temperature and stored at −20 °C as a protein isolate. The protein content was quantified using the BCA assay kit (Bicinchoninic Acid Protein Assay kit, Sigma, Lyon, France). Protein concentration was calculated by interpolation in a calibration curve prepared using a BSA standard at concentrations ranging from 0 to 6 mg/mL after standing at room temperature for 15 min. Every sample was measured by triplicate.

Equal amounts of protein samples were separated by SDS-polyacrylamide gel electrophoresis (SDS-PAGE). Laemmli buffer (62.5 mM of Tris-HCl (pH 6.8), 25% (*v/v*) of glycerol, 2% (*w/v*) of SDS and 0.01% (*w/v*) of bromophenol blue) was mixed at a 5% (*v/v*) with β-mercaptoethanol. Of this solution 15 μL was mixed with 15 μL of the sample and heated at 90 °C for 10 min in a Thermomixer Compact (Eppendorf AG, Hamburg, Germany).

Electrophoresis was carried out in a Mini-Protean cell (Bio-Rad, Hercules, CA, USA) by applying 70 V for 10 min and 150 V for 50 min. After separation, proteins were transferred to the polyvinylidene difluoride (PVDF) membrane (Amersham™ Hybond™, GE Healthcare). The membrane was incubated overnight at 4 °C with the polyclonal primary antibody directed against the Kv3.1b channel (Thermofisher Scientific, Waltham, MA, USA) and then after the secondary antibody, coupled with peroxidase (BioRad, Hercules, CA, USA) for 1 h in the dark at room temperature. Gels were scanned with a Fusion FX6 Imaging System (Vilber Smart imaging, Marne-la-Vallée, France).

### 3.3. Serotonin Quantification

Analyses were performed on a high-pressure liquid chromatography (HPLC) system on 1C11 cells supernatants. The standard serotonin (5-hydroxytryptamine creatinine sulphate complex, M, 405.40); di-sodium hydrogen phosphate, 2H_2_O and sodium dihydrogen phosphate, NaH_2_PO_4_ were purchased from Sigma (Saint Louis, MO USA). Acetic acid was purchased from Prolabo (Paris, France) and chloroform (CHCI, HPLC grade) purchased from Carlo Erba. Supernatants were frozen in a microcentrifuge tube at −80 °C.

For serotonin dosage with HPLC, supernatants were collected after culturing 1C11 cells in specific conditions. Then samples were homogenized by sonication 4 times (2 s pulse each time) on ice at 20 kHz. Of each supernatant 5 mL (ND(D4); D(D4); NDFlX (D4) and DFlX (D4)) were transferred in a specific heparinized vacutainer tube without gel. To each tube, 1.0 M of ascorbic acid, Merck (40 mg/4 mL sample) were added to each tube, and the samples were centrifuged at 10,000 rpm for 10 min at 4 °C. The supernatant was collected carefully and stored at −80 °C until use.

The HPLC analysis for the serotonin done using a BAS 460 MICROBORE-HPLC system with electrochemical detection (Bio-analytical Systems Inc., West Lafayette, IN, USA) together with a Uniget C-18 reverse phase microbore column as the stationary phase (BASi, Cat no. 8949). The mobile phase consisted of a buffer of 0.1 M monochloro acetic acid, 0.5 mM Na-EDTA, 0.15 g/L Na-octylsulfonate and 10 nM NaCl, pH 3.1 (all chemicals were from Sigma), acetonitrile and tetrahydrofuran (all solvents were from Fisher Scientific, Waltham, MA, USA) at a ratio of 94:3.5:0.7. The flow rate was 1.0 mL/min and the working electrode (Uniget 3 mm glassy carbon, BAS P/N MF-1003) was set at 550 mV vs. the Ag/Ag/Cl reference electrode. The detection gain was 1.0 nA, filter was 0.2 Hz and detection limit was set at 20 nA. Of the sample supernatant 5 μL was directly injected into the HPLC for analysis. Standard serotonin (Sigma, Lyon, France) was used to quantify and identify the peaks on the chromatographs. The retention time for serotonin was approximately 6.5 min. The detection limits for serotonin were determined by running the known concentrations of serotonin separately in the HPLC system under the set condition. For this purpose, a standard solution of 1000 μg/L is therefore made with pure serotonin and diluted accordingly to get the desired concentrations of the stock solutions for running in HPLC (100 μg/L and 200 μg/L).

#### 3.3.1. Electrophysiological Analysis

Potassium channels were recorded in 1C11 cell lines under different conditions, in proliferating cells in the absence or presence of precursors and in cells after differentiation with the patch clamp method.

Whole-cell recordings were realized at room temperature (24 °C), with an EPC-10 amplifier (HEKA Electronic, Lambrecht, Germany). The media used within the pipette were respectively (mM): 110 KCl, 5 NaCl, 2 MgCl_2_, 10 ethylene glycol-bis (2-aminoethylether)-*N*, *N*, *N*′, *N*′-tetraacetic acid (EGTA) and 5 mM HEPES and in the bath (mM): 100 *N*-methyl-D-Glucamine-Cl, 5 KCl, 2 MgCl_2_, 50 NaOH, 50 acetic acid and 5 HEPES at pH 7.3. Membrane currents were elicited from a holding potential of −80 mV, by depolarizations ranging from −120 to +60 mV. We used only cells with series resistance less than 5 MΩ for analysis. Patchmaster, Fitmaster (HEKA Electronic, Lambrecht, Germany) and IgorPro (WaveMetrics, Inc., OR, USA) software were used for data acquisition and analysis. Recording pipettes were from glass capillaries (Hematocrit, Modulohm A/S, DK). Their resistances were 1.5–4 MΩ and pulled with a PC-10 Narishige puller.

#### 3.3.2. Statistical Analysis

Statistical analysis was performed using Excel and the numerical data were expressed as mean ± SEM. Differences were tested using a Student’s *t*-test for unpaired data, assuming that the population follows a Gaussian distribution. Differences were assumed to be significant when *p* < 0.05.

## 4. Discussion and Conclusions

The present study provides evidence that fluoxetine affects Kv3.1b expression together with the secretion of serotonin during neuronal serotonergic 1C11 cell line development.

We show for the first time the presence of the Kv3.1b channel in the 1C11 cell line previously defined as a neuronal serotonergic cell line [35].

This study also provides for the first time a fingerprint of the potassium current expression in 1C11. It highlights how the bioactive compound, fluoxetine (an antidepressant drug) at 10 nM, affected Kv3.1b expression during 1C11 cell line differentiation in vitro. Serotonin receptors play a key role in depression disorders and are the first target of fluoxetine. Taken during depressive disorders, fluoxetine would exert therapeutic effects by enhancing serotonergic transmission, exclusively by inhibiting serotonin (5-hydroxytryptamine; 5HT) transporters with minimal or no effects on other neurotransmitter receptors [39,40].

1C11 is a serotonergic cell line used as an in vitro model for depression disorders to study the interaction between specific pharmacological compounds and their targets in relation to treatment. These cells secrete serotonin after differentiation because of 5HT receptors expression. We carried out a comparative study of the effect of fluoxetine on Kv3.1b channels and 5HT1b receptors in this serotonergic cell line as per this scheme: (1) the evaluation of Kv3.1b gene expression in the 1C11 cell line; (2) the protein level and the functional expression of potassium channels during 1C11 development in vitro; (3) the variation of Kv3.1b and 5HT1b mRNA expression in the 1C11 cell culture in vitro and upon fluoxetine and (4) the evaluation of the effect of fluoxetine on the serotonergic activity of 1C11 cells. The Kv3.1b channel expression was thus highly regulated during 1C11 cells differentiation in vitro. We found that the channel transcript expression in differentiated cells was significantly higher than in not differentiated cells. Fluoxetine acts differently as a function of the cell’s differentiation state. It up-regulated Kv3.1b mRNA expression in not-differentiated cells but it downregulated it when cells were differentiated. It seems that while fluoxetine enhances Kv3.1b mRNA synthesis in still not-differentiated cells, it was, paradoxically, slowing it down in mature cells. To elucidate the role of the Kv3.1b channel in the serotonergic 1C11 cell line, we characterized the transcripts level of other Kv channel types. We demonstrated that the expression of voltage gated potassium channels was modulated in 1C11 cells in vitro. While the Kv1.1, Kv1.3, Kv1.4 and Kv2.1 transcripts levels were reduced in differentiated cells, the Kv1.2 transcript level showed a small increase. We observed a remarkably higher increase in the level of Kv3.1b mRNA expression in differentiated cells in line with Grit Schaarschmidt’s [41] results on human neural progenitor cells, or hNPCs. However, hNPCs showed an increase in Kv1.1 and Kv2.1 contrary to the 1C11 cell line. Our finding is also supported by another study showing that mRNA levels of Kv3.1, Kir4.1 and Kir5.1 were significantly higher than those of other Kir and Kv channels [42]. Moreover, Kv3.1 was previously identified in oligodendrocyte progenitor cells as a critical Kv channel that controls the proliferation and myelination of axons [43].

The functional characterization of the potassium ionic currents, carried out in the whole cell configuration, revealed that 1C11D had higher current levels than 1C11ND. It is tempting to postulate that these currents are mainly carried out by Kv3.1 channels since they represent the main Kv channels in mature 1C11 cells. The Kv3.1b protein was present in 1C11 cells proliferating and differentiated but did not seem functional during proliferation as shown by the current recording. Kv3.1b channels should play a primordial role in 1C11 cell line development since they present a significant high expression of transcripts and proteins in differentiated cells. Further study is required, using selective inhibitors, to determine the exact contribution of Kv3.1 channels to the 1C11 potassium current.

Quantitative PCR analysis demonstrated that Kv3.1b transcript levels did not present a significant difference compared to 5HT1b transcript levels in 1C11D compared to 1C11ND cells. In fact, 1C11D cells are characterized by a high expression of serotonin receptors like 5HT1b and previous publications illustrate the ability of differentiated cells to secrete serotonin [36]. It is therefore possible to conclude that 1C11 serotonergic activity is dependent on cell differentiation but is not related to Kv3.1b firing activity. The central serotonin (5-hydroxytryptamine, 5-HT) system is involved in the vast majority of antidepressant treatments [44], including the selective serotonin reuptake inhibitors (SSRIs), such as fluoxetine (Prozac), which are the most widely used [45]. Previous studies have reported that antidepressant treatments affect the serotonergic system in the brain by inducing adaptive changes in various 5-HT receptors subtypes [46,47] and that chronic treatment with antidepressants selectively decreases the density of 5-HT_4_ receptors [48]. Another study performed in vitro, reported that low doses of fluoxetine up-regulated the expression of serotonin receptors was which were down-regulated by high doses of fluoxetine, and by serotonin in LS8 cells [49].

Under fluoxetine, we demonstrated increases in the Kv3.1b transcript level in proliferating cells and of Kv3.1b expression in differentiated cells.

For both 5-HTR and Kv3.1, fluoxetine modulates electrical activity [9,50], and the transmembrane protein expression of Kv3.1b is slightly upregulated in 1C11D as we demonstrated, but highly upregulated for 5HT1b. This strengthens the hypothesis that 5HTR is the main target of fluoxetine but does not exclude the possible impact on Kv3.1. Although the Kv3.1 function is not tightly related to the serotonergic activity consisting of serotonin synthesis and secretion, chronic use or high concentrations of Flx could affect the Kv3.1 channel function [51]. A rat model of depression showed that Kv3.1 expression decreased in the hippocampus after Chronic Mild Stress (CMS) [52], and that fluoxetine treatment induced a slight increase of Kv3.1 expression. This is consistent with our findings with fluoxetine treated 1C11D cells and supports our hypothesis that this cell line is a suitable model for studying the role of the Kv3.1 channel during depression and that its modulation could interfere with antidepressant compounds, and with the development and/or treatment of the disorder.

In the same conditions of 1C11 culture and analysis, our study showed that fluoxetine (at the nanomolar level) drastically decreased 5HTR transcript levels in differentiated cells and the amount of serotonin in the medium. This suggests that fluoxetine affects the kinetics of 1C11 cells differentiation and is explained by the 5HT1b transcripts decrease.

Most studies, report the reduction of the 5-HT1b receptor expression and function has been reported with SSRI, like fluoxetine in our case study. In fact, the role of 5-HT1b receptors in the regulation of 5-HT release is inhibitory. Upon binding to the 5-HT1b receptors, 5-HT inhibits the formation of cAMP and downstream cellular responses resulting in transmitter release decrease [53,54,55]. The results that we obtained shows the possible presence of some serotonin in the medium from the beginning leading to 5-HT reuptake inhibition by SERT when cells are not differentiated. More recent research supports that the 5-HT1b receptors can regulate serotonin transporter function, thus serving as an additional mechanism by which 5-HT1b modulates extracellular transmitter levels in serotonergic regions [56,57,58].

In conclusion, we provide the first experimental evidence that the 1C11 cell line expresses the transmembrane Kv3.1b channels, that play a primary role during 1C11 cell line development. Fluoxetine at 10 nM improved Kv3.1 transcript expression levels during the mitotic phase, corresponding to cell proliferation. It also slightly promoted Kv3.1b protein expression in differentiated cells. In addition, fluoxetine downregulated 5HTRs mRNA synthesis in differentiated cells and decreased the secretion of serotonin. It suggests that 10 nM fluoxetine slows down the kinetics of 1C11 cell differentiation leading to a decrease of the serotonin release and its amount in the medium.

## Figures and Tables

**Table 1:** Cells Treatment Conditions.

	DMEN + SVF 10%	dbcAMP	CCA	Fluoxetine 10 nM
1C11 ND (D4)	+	−	−	−
1C11 D (D4)	+	+	+	−
1C11 ND Flx (D4)	+	−	−	+
1C11 D Flx (D4)	+	+	+	+

ND (D4): Not Differentiated (dbcAMP -; CCA -), Day 4. D(D4): Differentiated with precursor (dbcAMP +; CCA +), Day 4. Flx: Fluoxetine 10 nM. dbcAMP: Dibutyryl AMP-cyclique. CCA: Cyclohexane carboxylic acid.

**Table 2:** The Primer Sequences used for PCR and qPCR Reactions.

Primer’s Name	Primer’s Sequence
F-hGAPDH	5′CGCTCTCTGCTCCTCCTGTT
R-hGAPDH	3′CCATGGTGTCTGAGCGATGT
Kv3.1-F	5′CTTTGCCTCCCTCTTCTTCATC
Kv3.1-R	3′TTCGGTCTTGTTCACGATGG
5HT1b-F	5′GGAGATGCTGGACTGCTTTG
5HT1b-R	3′GAGGAGCAGGGTGGGTAAAT
Kv1.1-F	GAAGAAGCTGAGTCGCACTTCTCCAG
Kv1.1-R	TTAAACATCGGTCAGGAGCTTGCTC
Kv1.2-F	GTCATCCGGTTGGTAAGAGTCTTTAG
Kv1.2-R	GTGTTAGCCAAGGTACAGTTGGCTG
Kv1.3-F	ATCTTCAAGCTCTCCCGACCA
Kv1.3-R	CGAATCACCATATACTCCGAC
Kv1.4-F	GCTCACTCCAGGGCAGCTGCAGCTGCTGCT
Kv1.4-R	TCACGCATGCTGGCTCTTAGGGTGTGGCCC
Kv2.1-F	CTCCACCATTGCCCTGTC
Kv2.1-R	TCCGCTTGATTGCTTTCTC

F: Forward; R: Reverse.
